# A Novel Protective Function of 5-Methoxytryptophan in Vascular Injury

**DOI:** 10.1038/srep25374

**Published:** 2016-05-05

**Authors:** Yen-Chun Ho, Meng-Ling Wu, Chen-Hsuan Su, Chung-Huang Chen, Hua-Hui Ho, Guan-Lin Lee, Wei-Shiang Lin, Wen-Yu Lin, Yu-Juei Hsu, Cheng-Chin Kuo, Kenneth K. Wu, Shaw-Fang Yet

**Affiliations:** 1 Institute of Cellular and System Medicine, National Health Research Institutes, Zhunan, Taiwan; 2 Graduate Institute of Life Sciences, National Defense Medical Center, Taipei, Taiwan; 3 National Institute of Environmental Health Sciences, National Health Research Institutes, Zhunan, Taiwan; 4 Division of Cardiology, Department of Medicine, Tri-Service General Hospital, Taipei, Taiwan; 5 Division of Nephrology, Department of Medicine, Tri-Service General Hospital, Taipei, Taiwan; 6 Metabolomic Research Center and Graduate Institute of Basic Medical Science, China Medical University, Taichung, Taiwan; 7 Department of Medical Sciences and Institute of Biotechnology, National Tsing Hua University, Hsinchu, Taiwan

## Abstract

5-Methoxytryptophan (5-MTP), a 5-methoxyindole metabolite of tryptophan metabolism, was recently shown to suppress inflammatory mediator-induced cancer cell proliferation and migration. However, the role of 5-MTP in vascular disease is unknown. In this study, we investigated whether 5-MTP protects against vascular remodeling following arterial injury. Measurements of serum 5-MTP levels in healthy subjects and patients with coronary artery disease (CAD) showed that serum 5-MTP concentrations were inversely correlated with CAD. To test the role of 5-MTP in occlusive vascular disease, we subjected mice to a carotid artery ligation model of neointima formation and treated mice with vehicle or 5-MTP. Compared with vehicle-treated mice, 5-MTP significantly reduced intimal thickening by 40% 4 weeks after ligation. BrdU incorporation assays revealed that 5-MTP significantly reduced VSMC proliferation both *in vivo* and *in vitro*. Furthermore, 5-MTP reduced endothelial loss and detachment, ICAM-1 and VCAM-1 expressions, and inflammatory cell infiltration in the ligated arterial wall, suggesting attenuation of endothelial dysfunction. Signaling pathway analysis indicated that 5-MTP mediated its effects predominantly via suppressing p38 MAPK signaling in endothelial and VSMCs. Our data demonstrate a novel vascular protective function of 5-MTP against arterial injury-induced intimal hyperplasia. 5-MTP might be a therapeutic target for preventing and/or treating vascular remodeling.

Occlusive vascular disease is the main underlying cause of cardiovascular disease, which is a major health issue worldwide with significant morbidity and mortality^1^. In normal adult blood vessels, vascular smooth muscle cells (VSMCs) exhibit a differentiated contractile phenotype and proliferate at a low rate. In response to injury, VSMCs change from a quiescent contractile phenotype to a synthetic phenotype, acquiring the ability to migrate and proliferate at a high rate^2^,^3^. The migration and proliferation of VSMCs from the media into the intima contribute to arterial intimal thickening and subsequent arteriosclerosis^2^,^4^. In addition to VSMC proliferation and migration, inflammation may play a critical role in the development of stenosis/restenosis^5^. In response to pathological stimulation, VSMCs can exhibit an inflammatory phenotype, expressing adhesion molecules such as vascular adhesion molecule-1 (VCAM-1), allowing adhesion of monocytes and lymphocytes within the vessel wall^3^. Moreover, endothelium of the vascular luminal lining normally protects blood vessels from adhesion of leukocytes. However, pathological stimulation and injury activate endothelium, resulting in enhanced expression of adhesion molecules including intercellular adhesion molecule-1 (ICAM-1) and VCAM-1, allowing adhesion and transmigration of leukocytes into the artery, leading to VSMC phenotypic changes and subsequent vascular modeling^6^. Therefore, vascular disease is regarded as an inflammatory disease^6^,^7^,^8^. As such, protecting against vascular inflammation, VSMC proliferation and migration, and endothelial dysfunction is critical in attenuating vascular lesion formation after pathological stress.

5-Methoxytryptophan (5-MTP) is a 5-methoxyindole metabolite of tryptophan metabolism^9^,^10^. 5-Methoxyindoles such as melatonin, 5-methoxytryptamine, and 5-methoxytryptophol participate in the control of circadian rhythm in the pineal gland and retina^11^,^12^,^13^,^14^,^15^ and are regarded as neuromodulators^16^,^17^. However, unlike these 5-methoxyindoles, the physiological function of 5-MTP is unclear, although it has been implicated a role in the pineal gland and retina^11^,^18^. Interestingly, we recently identified a function of 5-MTP in tissues other than pineal gland and retina. We found that 5-MTP could suppress inflammatory mediator-induced cancer cell proliferation and migration, thereby reducing tumor growth and metastasis^9^,^10^. Another study showed that 5-MTP is able to protect cardiomyocytes from H_2_O_2_-induced oxidative injury^19^. However, the role of 5-MTP in vascular disease is unknown. The pathogenic VSMC migration and proliferation are somewhat similar to that of cancer cell growth and invasion. We hypothesized that 5-MTP might protect against vascular injury-induced medial VSMC proliferation and migration and subsequent neointima formation and occlusive vascular remodeling.

The goal of the present study was to investigate whether 5-MTP has a role in vascular disease. Measurements of serum 5-MTP levels in healthy subjects and patients with coronary artery disease (CAD) showed that serum 5-MTP concentrations are inversely correlated with CAD. In a mouse carotid artery ligation model, we showed that 5-MTP significantly reduced neointima area after ligation, likely through attenuating VSMC proliferation and migration. In addition, 5-MTP decreased ligation-induced endothelial adhesion molecule expressions and inflammatory cell infiltration. Our results indicate that in response to arterial injury, 5-MTP protects against endothelial dysfunction and VSMC proliferation and migration, thereby reducing intimal hyperplasia. Taken together, 5-MTP might be a therapeutic target for preventing and/or treating vascular remodeling.

## Results

### Serum 5-MTP concentrations are inversely correlated with coronary artery disease

**Figure 1:**
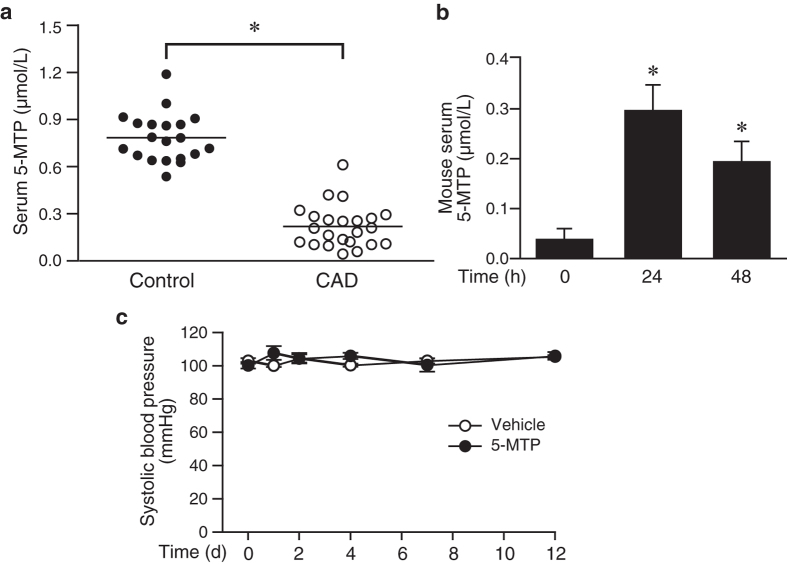
Serum 5-MTP concentrations and effect of 5-MTP on mouse blood pressure. (**a**) Twenty-three CAD patients and 20 control subjects without CAD and known systemic disease were included in the study. Blood was drawn from patients prior to angiography and serum 5-MTP levels measured by competitive ELISA assays and expressed as μmol/L. *P < 0.0001 vs. control. (**b**) Approximately 12 weeks old C57BL/6 wild-type mice were injected with 100 mg/kg of 5-MTP by intraperitoneal injection. Blood was collected before (n = 8), after 24 h (n = 4), and 48 h (n = 4) of 5-MTP injections. Serum levels of 5-MTP were measured by competitive ELISA assays. Values are mean ± SE. *P < 0.05 vs. control before exogenous 5-MTP administrations. (**c**) Systolic blood pressure (SBP) of mice at baseline and at several time points after initial vehicle (n = 6) or 5-MTP (n = 6) injection (100 mg/kg, 3 times a week).

To investigate the clinical relevance of 5-MTP in human CAD, we measured serum 5-MTP levels in control subjects and CAD patients. Control subjects did not have any coronary vessel and known systemic disease. The presence of CAD was confirmed by coronary angiography and CAD was defined as more than 50% angiographic diameter stenosis in one or more coronary arteries. Patients who had diabetes, hypertension, or stroke were excluded from the study. Twenty control subjects (10 males and 10 females, mean age 59 ± 7 years) and 23 CAD patients (17 males and 6 females, mean age 61 ± 10 years) were included in the study. The body mass index and blood pressure were not different between control and CAD groups (Table 1). Serum was obtained from blood drawn from controls or patients prior to angiography for biochemical analysis (Table 1). Although serum creatinine levels were higher in CAD group but it did not reach a statistical significance. Patients in CAD group had higher serum glucose levels than those in the control group but they were not diabetic. Cholesterol and triglyceride levels were within normal ranges in both groups. Importantly, serum IL-6 levels, an indicator of inflammation, were significantly higher in CAD group (Table 1). Measurements of 5-MTP with enzyme-immunoassays revealed that the mean serum 5-MTP levels from controls were 0.78 ± 0.15 μmol/L (Fig. 1a). In contrast, serum 5-MTP concentrations of CAD patients were significantly reduced to 0.22 ± 0.13 μmol/L (Fig. 1a, *P* < 0.0001 vs. control). These results indicate that serum 5-MTP concentrations are inversely correlated with CAD, implicating a potential role of 5-MTP in occlusive vascular disease.

### 5-MTP reduces neointima formation in a mouse carotid artery ligation model

**Figure 2:**
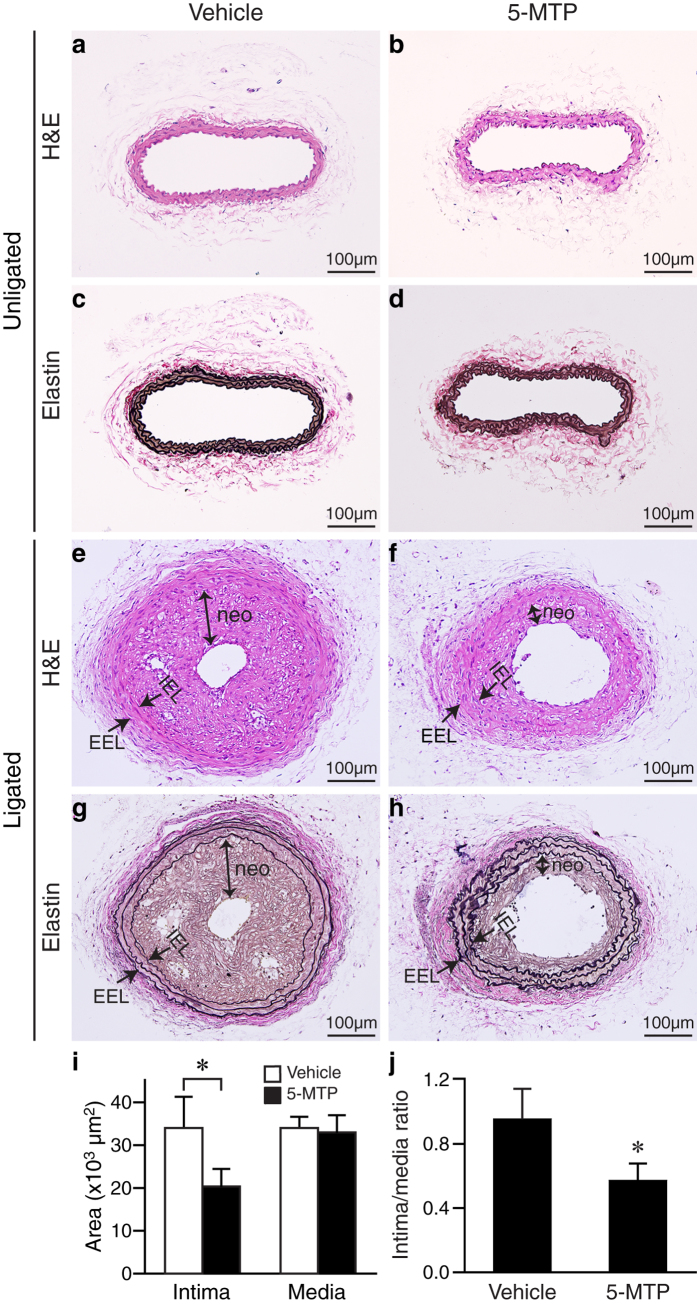
5-MTP reduces intimal thickening in response to arterial injury. Mice were subjected to carotid artery ligation, followed by IP injection of vehicle or 5-MTP. Unligated (**a**–**d**) and ligated (**e**–**h**) carotid arteries were harvested 4 weeks later. Vessel sections were stained with H&E (**a,b,e,f**) or Verhoeff’s staining for elastin (black) (**c,d,g,h**). Representative sections are shown. IEL, internal elastic lamina; EEL, external elastic lamina; neo, neointima. (**i**) Quantitative morphometric analysis of intimal and medial area in vehicle-treated (n = 9) and 5-MTP-treated mice (n = 12). *P < 0.05 vs. vehicle. (**j**) Compared with vehicle-treated mice (n = 9), 5-MTP reduced intima/media area ratio (n = 12, *P < 0.05).

We next wanted to investigate the role of 5-MTP in vascular remodeling. To this end, we first examined mouse serum 5-MTP levels. ELISA assays showed that mouse has an endogenous serum concentration of 0.04 ± 0.02 μmol/L (n = 8, Fig. 1b). Intraperitoneal (IP) injection of 5-MTP (100 mg/kg, a dose used in a previous study^9^) increased mouse serum 5-MTP levels by 7.5-fold to 0.30 ± 0.05 μmol/L (n = 4, *P* < 0.05 vs. control) at 24 h and somewhat decreased to 0.20 ± 0.04 μmol/L (n = 4) at 48 h (*P* < 0.05 vs. control) (Fig. 1b). As blood pressure affects vascular remodeling, we evaluated whether 5-MTP has an effect on blood pressure. Systolic blood pressure was not different between vehicle (n = 6) and 5-MTP (n = 6) groups at baseline or at several time points following initial injection (Fig. 1c), indicating 5-MTP does not affect blood pressure. We thus subjected mice to a neointima formation model of carotid artery ligation and treated mice with vehicle or 5-MTP (3 times/week). Before ligation, the body weight was similar between vehicle (26.0 ± 0.9 g, n = 9) and 5-MTP groups (26.2 ± 0.5 g, n = 12). Compared with vehicle controls, administration of 5-MTP did not affect body weight 4 weeks later when carotid arteries were harvested (26.0 ± 0.4 g vs. vehicle group 25.9 ± 0.9 g), suggesting no adverse effect of 5-MTP. H&E staining of vessel sections showed patent unligated arteries (Fig. 2a,b). Verhoeff’s staining, to delineate elastin layers, revealed no intimal hyperplasia in the unligated control arteries (Fig. 2c,d). Ligation induced robust neointima formation in vehicle-treated carotid arteries (Fig. 2e,g). In contrast, neointima was much smaller in ligated carotids from 5-MTP-treated mice (Fig. 2f,h). Morphometric analysis showed that despite similar medial area between vehicle- and 5-MTP-treated groups (34030 ± 2608 vs. 33013 ± 4022 μm^2^, respectively), 5-MTP significantly reduced neointimal area from 34049 ± 7292 μm^2^ (vehicle group, n = 9) to 20320 ± 4167 μm^2^ (n = 12, *P* < 0.05) (Fig. 2i). In addition, 5-MTP significantly decreased intima/media ratio from 0.96 ± 0.19 of vehicle group to 0.58 ± 0.11 of 5-MTP group (*P* < 0.05; Fig. 2j).

### 5-MTP inhibits smooth muscle cell proliferation after arterial injury

**Figure 3:**
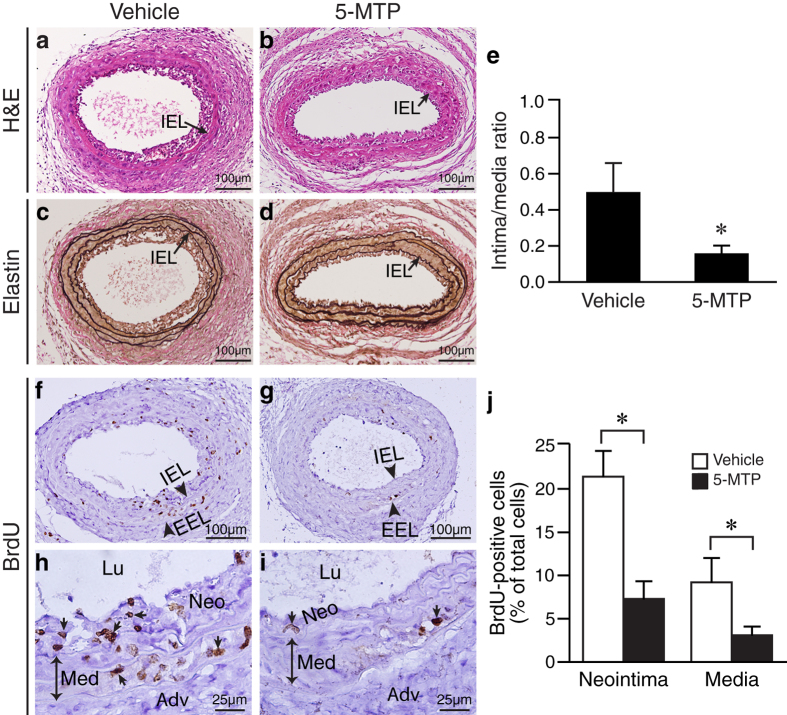
5-MTP inhibits intimal hyperplasia and VSMC proliferation in the injured arteries. Mice were subjected to carotid artery ligation, followed by IP injection of vehicle (PBS, n = 6) or 5-MTP (n = 9). Carotid arteries were harvested one week after ligation. Mice were injected twice with BrdU at 16–18 h and 1–2 h before harvest. (**a,b**) Vessel sections were stained with H&E or (**c,d**) Verhoeff’s staining for elastin (black). Representative sections are shown. IEL, internal elastic lamina. (**e**) Quantitative morphometric analysis of intimal area/media area ratio in 1-week ligated carotid arteries (*P < 0.05). (**f**–**i**) Vessel sections were stained with a monoclonal BrdU antibody to detect proliferating cells and counterstained with hematoxylin. Small arrows indicate BrdU-positive brown nuclei. IEL, internal elastic lamina; EEL, external elastic lamina; Neo, neointima; Med, medial layer; Adv, adventitia; Lu, lumen. (**j**) Immunostained positive cells were quantified as the number of positive cells divided by total number of nuclei in the neointima or media, and expressed as % of total cells. *P < 0.05 vs. vehicle group.

We next investigated the mechanisms by which 5-MTP protects against neointima formaton. Proliferation and migration of VSMCs from the media into intima contributes to arterial intimal thickening and subsequent arteriosclerosis^2^,^4^. In the carotid artery ligation model, VSMC proliferation is a prominent feature of neointima formation and the index is high in the media and intima one week after ligation^20^,^21^. Thus, we examined neointima formation at this time point. As reported previously^22^, one week after ligation neointima was evident, although small, in vehicle-treated mice (Fig. 3a,c). In comparison, 5-MTP substantially reduced neointimal area (Fig. 3b,d) and significantly decreased the intima/media ratio from 0.50 ± 0.16 of vehicle group (n = 6) to 0.16 ± 0.04 (n = 9; *P* < 0.05; Fig. 3e).

To examine whether the reduced neointima formation by 5-MTP was due to decreased proliferation, mice were injected with BrdU at 16–18 h and 1–2 h before harvest. We then assessed BrdU incorporations by immunostaining vessel sections with BrdU antibodies. Many BrdU-positive cells were detected in the neointima and media from vehicle-treated ligated carotids (Fig. 3f,h) whereas 5-MTP treatment decreased BrdU-positive cell number in the neointima and media (Fig. 3g,i). Quantitative analysis showed that proliferation index was much higher in vehicle-treated vessels with 21.4 ± 3.0% BrdU incorporation in the neointima and 9.2 ± 2.9% in the media (Fig. 3j, white bars). In 5-MTP-treated vessels, we observed 7.3 ± 2.1% of BrdU incorporation in the neointima (*P* < 0.05 vs. vehicle group) and 3.1 ± 1.0% in the media (*P* < 0.05 vs. vehicle group; Fig. 3j, black bars), indicating reduced cellular proliferation in 5-MTP-treated mice in both neointima and media.

### 5-MTP inhibits IL-1β-induced smooth muscle cell proliferation and migration

**Figure 4:**
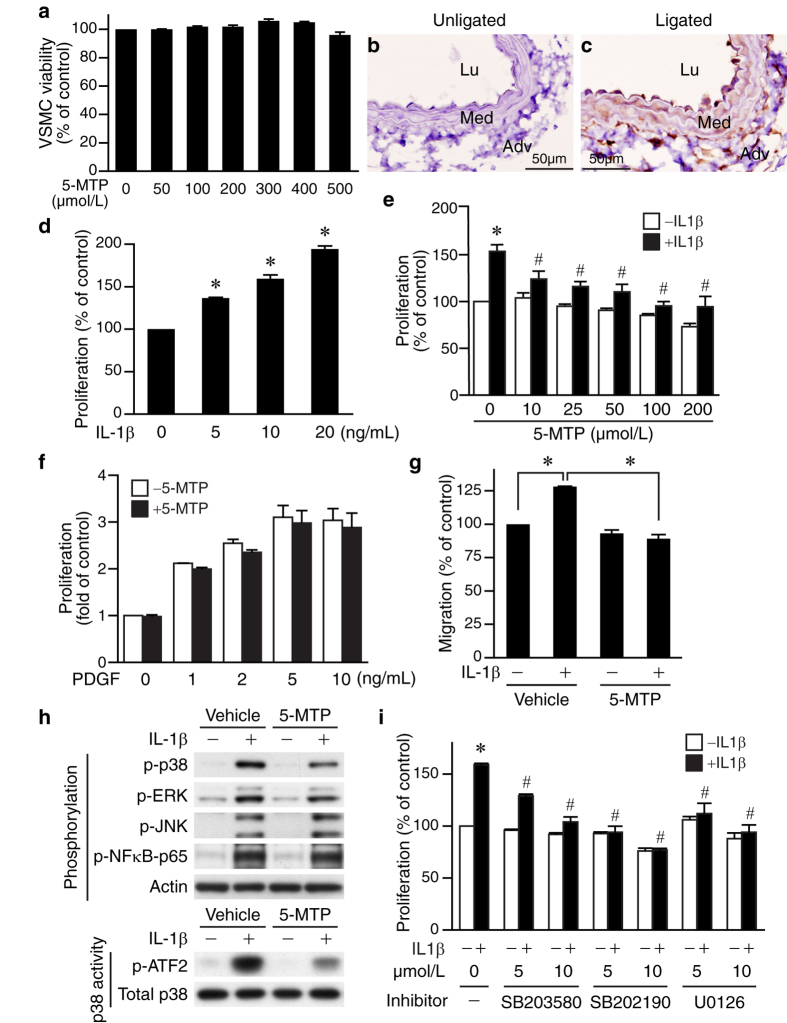
5-MTP suppresses VSMC proliferation via p38 MAPK and ERK pathway. (**a**) MTT assays were performed to assess the effects of 5-MTP on VSMC viability. Immunohistochemistry of (**b**) unligated and (**c**) 4 d-ligated vessel sections were performed to detect IL-1β expression (brown). (**d**) Serum-starved VSMCs were treated with different doses of IL-1β for 24 h, proliferation then assessed by BrdU incorporations and normalized to control without IL-1β stimulation. *P < 0.05 vs. control. (**e**) Serum-starved VSMCs (in the absence or presence of different doses of 5-MTP) were treated with IL-1β for 24 h and proliferation assessed and normalized to control without IL-1β stimulation. *P < 0.05 vs. control; ^#^*P* < 0.05 vs. IL-1β-treated but without 5-MTP. (**f**) Serum-starved VSMCs were stimulated with different concentrations of PDGF-BB in the presence or absence of 5-MTP (100 μmol/L) for 24 h and proliferation assessed. (**g**) Serum-starved VSMCs (in the absence or presence of 5-MTP) were treated with IL-1β for 24 h, and migration assays performed. VSMCs migrating through the filters were quantified after 4 h. *P < 0.05. (**h**) Upper panel, VSMCs were serum starved in the absence or presence of 5-MTP, stimulated with or without IL-1β for 15 min, and total proteins isolated for Western blotting to detect phosphorylations of p38 MAPK, ERK1/2, JNK, and NFκB-p65 (Ser536). To verify equal loading, the blots were probed with a pan-actin antibody. A representative of 3 independent experiments is shown. Lower panel, p38 MAPK activity of VSMCs was measured using a p38 MAP kinase assay kit. Phosphorylation of ATF2 indicates p38 MAPK activity. Aliquots of cell lysates were subjected to Western blotting with p38 antibody to verify equivalent amount of protein for activity assessment. A representative of 3 independent experiments is shown. (**i)** VSMCs were pretreated with p38 MAPK inhibitor SB203580 or SB202190, or MEK inhibitor U0126 30 min prior to IL-1β stimulation. Proliferation was then assessed 24 h later. *P < 0.05 vs. control without IL-1β; ^#^*P* < 0.05 vs. IL-1β-treated but without inhibitor. Values are mean ± SE of at least three experiments.

To further confirm that the reduced intimal hyperplasia was due to decreased VSMC proliferation but not increased cell death by 5-MTP, we treated VSMCs with 5-MTP and assessed cell viability. Increasing concentrations of 5-MTP, even up to 500 μmol/L, did not exert any cytotoxic effect (Fig. 4a), ruling out the possibility that 5-MTP affected cell viability. Proinflammatory cytokine IL-1β has been shown to play a critical role in neointima formation following vessel injury, such as balloon angioplasty^23^, vein graft remodeling^24^, or carotid artery ligation^25^,^26^. Indeed, we found that carotid ligation induced IL-1β expression in the vessel wall (Fig. 4b,c). Furthermore, IL-1β stimulated VSMC proliferation in a concentration-dependent manner (Fig. 4d). Given the critical role of IL-1β in vessel injury and VSMC proliferation, we pretreated VSMCs with different concentrations of 5-MTP, stimulated with or without IL-1β, and then assessed proliferation. In the absence of IL-1β, 5-MTP did not affect VSMC proliferation (Fig. 4e). In contrast, 5-MTP significantly decreased IL-1β-induced proliferation (Fig. 4e). Intriguingly, 5-MTP did not affect growth factor PDGF-BB-induced VSMC proliferation (Fig. 4f), suggesting 5-MTP preferentially affects IL-1β-mediated effects. In addition to proliferation, VSMC migration contributes to intimal hyperplasia. We then performed migration assays to test whether 5-MTP affects VSMC migration in response to chemoattractant stimulation. Interestingly, IL-1β treatment increased VSMC migration while 5-MTP abrogated the enhanced migration of IL-1β (Fig. 4g). Taken together, these results indicate that 5-MTP attenuates IL-1β-induced VSMC proliferation and migration and reduces neointima formation following arterial injury.

### 5-MTP inhibits IL-1β-induced VSMC proliferation via suppressing MAPK activation

We next investigated potential mechanisms that mediate the suppressive effect of 5-MTP on IL-1β-induced VSMC proliferation. Mitogen-activated protein kinases (MAPKs) such as p38 MAPK and ERK1/2 participate in mediating VSMC proliferation regulated by various mediators^27^,^28^,^29^,^30^,^31^ and in the pathogenesis of neointimal hyperplasia after vascular injury^32^,^33^. Thus, we examined the involvement of MAPK signaling pathways in IL-1β-induced VSMC proliferation. Indeed, IL-1β induced phosphorylation of p38 MAPK, ERK1/2, JNK, and NFκB-p65 (Fig. 4h, upper panel). Interestingly, 5-MTP reduced p38 MAPK and ERK1/2 phosphorylation, particularly that of p38, but not JNK or NFκB-p65 phosphorylation (Fig. 4h). To ensure that the reduced p38 phosphorylation reflected on reduced activity, we immunoprecipitated phosphorylated p38 first and then used ATF2 as a substrate to measure p38 MAPK activity. 5-MTP attenuated ATF2 phosphorylation, indicating reduced p38 MAPK activity (Fig. 4h, lower panel). Furthermore, both p38 inhibitors SB203580 and SB202190 abrogated IL-1β-induced VSMC proliferation (Fig. 4i). Similarly, blocking ERK1/2 signaling by inhibitor U0126 diminished IL-1β-induced VSMC proliferation (Fig. 4i). These results suggest that the suppressive effect of 5-MTP on IL-1β-induced VSMC proliferation is mediated primarily through attenuated activation of p38 MAPK and ERK1/2 signaling.

### 5-MTP reduces carotid artery endothelial damage after ligation

**Figure 5:**
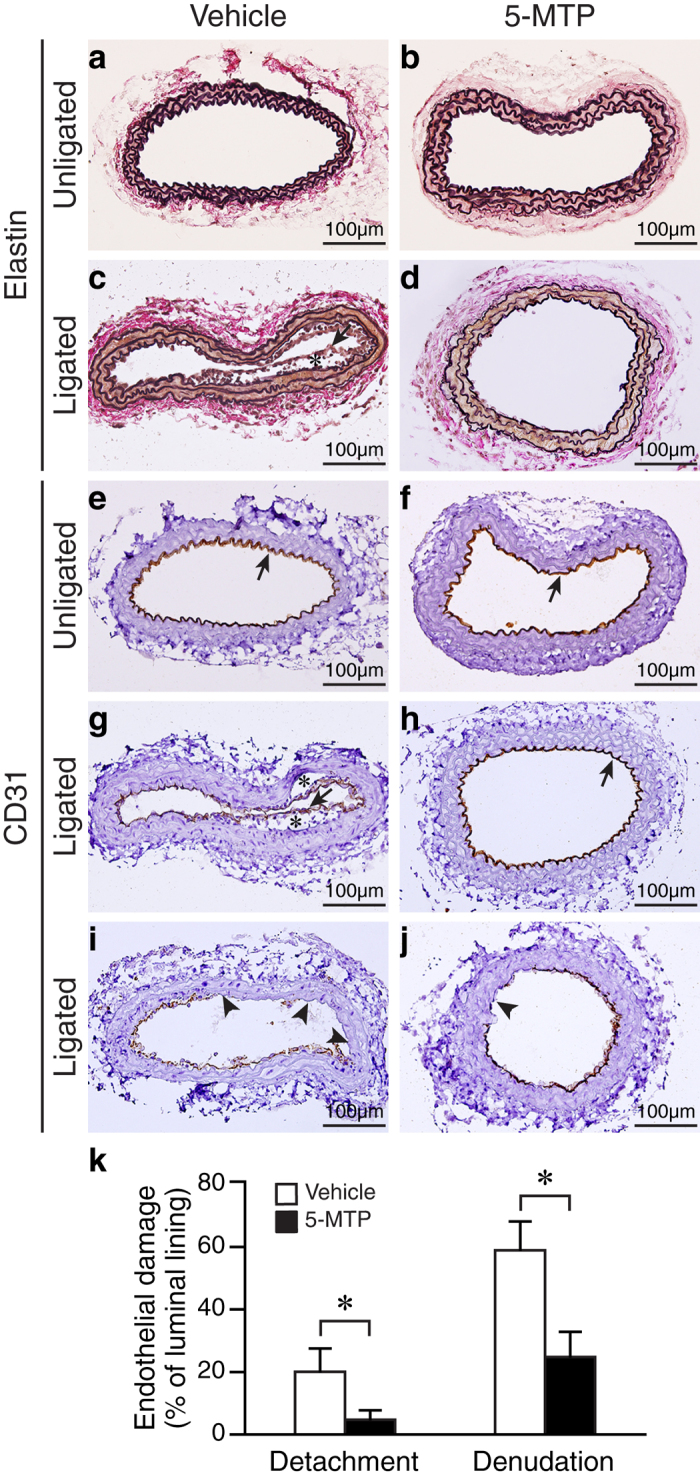
5-MTP protects against endothelial damage of mouse carotid arteries after ligation. Mice were subjected to carotid artery ligation surgery, followed by IP injection of vehicle (n = 10) or 5-MTP (n = 9). The carotid arteries were harvested 4 d later for histological analysis. (**a**–**d**) Vessel sections were stained with Verhoeff’s stain for elastin. Unligated vehicle-treated (**a**) and 5-MTP-treated (**b**) arteries. (**c**) Detachment of endothelium (arrow) in the vehicle-treated ligated arteries. *Enlarged subendothelial space. (**d**) 5-MTP treatment preserves vessel morphology in the ligated arteries. (**e**–**j**) Vessel sections were stained with endothelial cell marker CD31 antibody to identify endothelium. Arrow indicates endothelium. (**g**) Detachment of endothelium in the vehicle-treated ligated arteries. *Enlarged subendothelial space. (**h**) Intact endothelium in the 5-MTP-treated ligated arteries. (**i**) Loss of endothelium (arrowheads) in the vehicle-treated ligated arteries. (**j**) Small denuded area (arrowhead) in the 5-MTP-treated ligated arteries. (**k**) Quantitative morphometric analysis was performed to determine percentage of endothelial detachment and denudation from vehicle- and 5-MTP-treated group (*P < 0.05, n = 5 each group).

Endothelial damage/dysfunction is one of the initial events and an underlying cause of vascular remodeling/disease. Therefore, we hypothesized that in addition to VSMCs, 5-MTP might also protect endothelium against injury and subsequent neointima formation. To test our hypothesis, we examined carotid arteries at an early time point (4 d) after ligation. In the carotid artery ligation model, although endothelium is not removed it is frequently found to be detached from the underlying internal elastic lamina at an early time point^20^. Elastin staining showed intact unligated arteries (Fig. 5a,b). However, 4 d after ligation we observed endothelium detachment in vehicle- but not 5-MTP-treated ligated arteries (Fig. 5c,d). Endothelial marker CD31 staining demonstrated intact endothelium in the unligated arteries (Fig. 5e,f). In contrast, detachment and loss of endothelium in vehicle-treated ligated arteries (Fig. 5g,i) were obvious while endothelium was relatively intact or with minor damage from mice treated with 5-MTP (Fig. 5h,j). Quantitative analysis revealed that ligation caused 20.1 ± 7.4% endothelial detachment and 58.9 ± 9.3% denudation whereas 5-MTP reduced endothelial detachment and denudation to 4.7 ± 3.1% and 24.7 ± 8.1%, respectively (*P* < 0.05 vs. vehicle group, n = 5 each group) (Fig. 5k), indicating 5-MTP protects against endothelial injury.

### 5-MTP decreases endothelial ICAM-1 and VCAM-1 expressions in the ligated arteries

**Figure 6:**
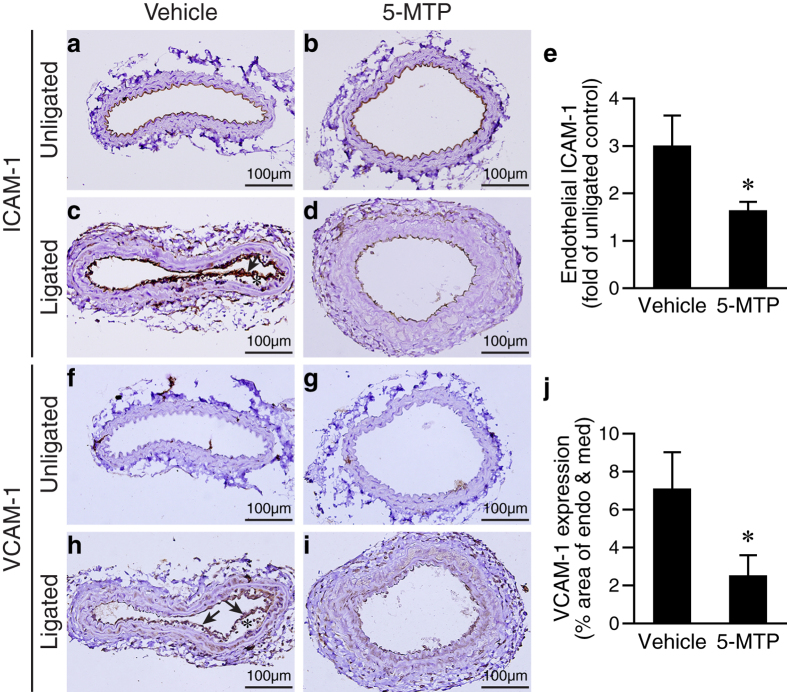
5-MTP suppresses injury-induced adhesion molecule expressions in endothelium. Vessel sections of carotid arteries harvested 4 d after ligation were stained for adhesion molecule expressions. ICAM-1 expression (brown) in the endothelium of unligated (**a,b**) and ligated (**c,d**) arteries. (**e**) ICAM-1 expression was quantified and expressed as fold of unligated control (*P < 0.05, n = 5 each group). (**f,g**) VCAM-1 is not detectable in the unligated arteries. (**h**) Enhanced expression of VCAM-1 in the vessel wall of vehicle-treated ligated arteries. Arrow, endothelium; *subendothelial space. (**i**) Low level expression of VCAM-1 in the 5-MTP-treated ligated arteries. (**j**) VCAM-1 expression was measured by colorimetric analysis and expressed as % area of endothelium and media (*P < 0.05, n = 5 each group).

In light of the apparent morphological improvement of ligated carotid endothelium in 5-MTP-treated mice, we next determined whether 5-MTP treatment protects against endothelial activation/dysfunction. Immunohistochemistry revealed that adhesion molecule ICAM-1 was normally expressed in the endothelium of unligated control arteries (Fig. 6a,b). Despite that ligation resulted in certain degree of endothelial loss (Fig. 5i,j), ligation enhanced ICAM-1 expression by 3.0 ± 0.6 fold in the remaining endothelium (Fig. 6c,e). In comparison, 5-MTP treatment significantly reduced ICAM-1 expression to 1.6 ± 0.2 fold of control (*P* < 0.05 vs. vehicle group, n = 5 each group) (Fig. 6d,e). Another adhesion molecule VCAM-1 was not expressed in the control unligated arteries (Fig. 6f,g), but markedly induced in the vehicle-treated ligated arteries, not only in endothelium but also in medial layer (7.1 ± 1.9% area of endothelium and media; Fig. 6h,j). The upregulation of VCAM-1 in medial layer after injury is consistent with the notion that VCAM-1 induction in VSMCs has a role in vascular disease^3^,^34^. Importantly, 5-MTP decreased ligation-induced VCAM-1 expression in the endothelium and medial layer to 2.5 ± 1.1% (*P* < 0.05 vs. vehicle group, n = 5 each group) (Fig. 6i,j).

**Figure 7:**
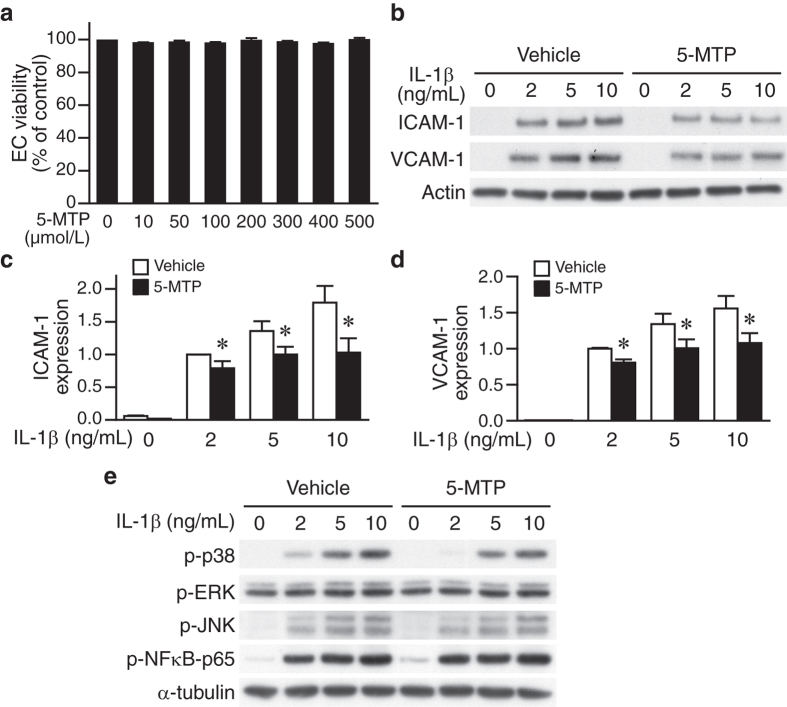
5-MTP suppresses IL-1β-induced expressions of adhesion molecules and p38 MAPK activation in endothelial cells. (**a)** Human umbilical vein endothelial cells (HUVECs) were seeded at a density of 1.6 × 10^4^ cells per well of 96-well plate. After overnight incubation, HUVECs were treated with indicated concentrations of 5-MTP in culture medium for 24 h, and cell viability assessed by MTT assays. (**b**) HUVECs were pretreated with vehicle or 5-MTP, followed by stimulation with the indicated dose of IL-1β for 24 h. Western blot analyses were performed to detect ICAM-1 and VCAM-1 levels. Blots were subsequently probed with a pan-actin antibody for loading control. A representative of 3 independent experiments is shown. (**c**) ICAM-1 and (**d**) VCAM-1 induction is expressed relative to vehicle with 2 ng/mL IL-1β stimulation (because of the very low level expressions without IL-1β stimulation). Values are mean ± SE of 4 experiments. *P < 0.05 vs. vehicle group. (**e**) HUVECs were pretreated with vehicle or 5-MTP, stimulated with different concentrations of IL-1β for 15 min. Western blotting was performed to detect phosphorylations of p38 MAPK, ERK1/2, JNK, and NFκB-p65 (Ser536). To verify equal loading, the blots were probed with a α-tubulin antibody. A representative of 3 independent experiments is shown.

To further characterize the effect of 5-MTP on adhesion molecule expressions in endothelial cells, we first examined the effect of 5-MTP on endothelial cell viability. Increasing concentrations of 5-MTP (10–500 μmol/L) did not exert any cytotoxic effect (Fig. 7a). As IL-1β plays a key role in modulating neointima formation following carotid artery ligation^25^,^26^, we examined ICAM-1 and VCAM-1 expression levels in endothelial cells after IL-1β stimulation. IL-1β increased ICAM-1 and VCAM-1 expressions at various concentrations in vehicle-treated endothelial cells (Fig. 7b–d). Importantly, consistent with *in vivo* findings, 5-MTP significantly attenuated IL-1β-induced ICAM-1 and VCAM-1 expressions (Fig. 7b–d).

We next investigated potential signaling mechanisms that mediate the inhibitory effect of 5-MTP on IL-1β-induced adhesion molecule expressions. As with VSMCs, IL-1β substantially induced phosphorylation of p38 MAPK, JNK, and NFκB-p65, but rather moderate of ERK1/2, in endothelial cells (Fig. 7e). Interestingly, 5-MTP predominantly reduced phosphorylation of p38 MAPK, but did not have much effect on ERK1/2, JNK, or NFκB-p65 phosphorylations (Fig. 7e). These results suggest that the decreases of endothelial adhesion molecule expressions by 5-MTP might be mediated through attenuated p38 MAPK activation.

### 5-MTP reduces inflammatory cell adhesion and infiltration to the vessel wall after carotid artery ligation

**Figure 8:**
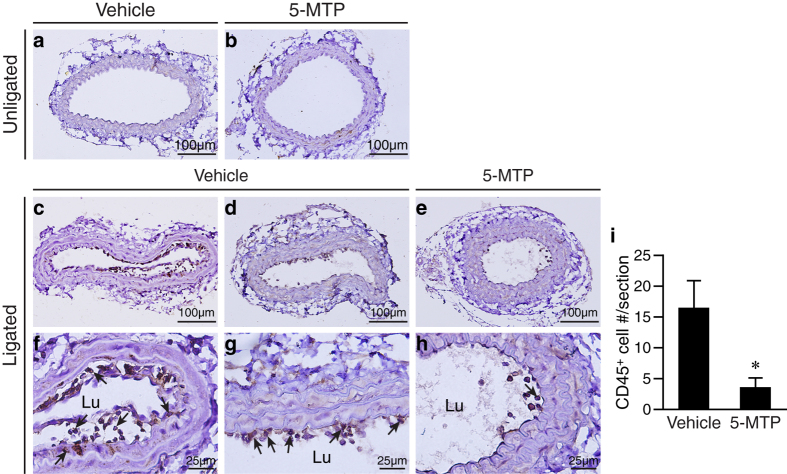
5-MTP attenuates vascular injury-induced inflammatory cell adhesion and infiltration. Mice were subjected to carotid artery ligation, followed by IP injection of vehicle (n = 10) or 5-MTP (n = 9). The unligated (**a,b**) and ligated (**c–h**) arteries were harvested 4 d after surgery for histological analysis. Vessel sections were stained with CD45 antibodies for inflammatory cells and representative sections are shown. No CD45-positive cells were detectable in (**a**) vehicle-treated unligated and (**b**) 5-MTP-treated unligated arteries. (**c**) Many CD45-positive (brown color) cells in the vessel wall of vehicle-treated ligated arteries. (**d**) Many CD45-positive cells on the luminal surface of vehicle-treated ligated arteries. (**e**) Few CD45-positive cells are detected in 5-MTP-treated ligated vessels. (**f**–**h**) Higher magnification of (**a**–**c**), respectively. (**f**) Arrows indicate CD45-positive cells adhered to the endothelium, infiltrated into subendothelial space, and medial layer. (**g**) Arrows indicate CD45-positive cells adhered to the luminal surface. (**h**) Few CD45-positive cells adhered to the luminal surface of ligated arteries from 5-MTP-treated mice. Lu, lumen. (**i**) Immunostained CD45-positive cells were quantified as the number of positive cells per vessel section. *P < 0.05 vs. vehicle group (n = 5 each group).

ICAM-1 and VCAM-1 are important for leukocyte adhesion and trans-endothelial migration^35^. Given that 5-MTP attenuated the induction of ICAM-1 and VCAM-1 after arterial injury, 5-MTP might attenuate inflammatory cell adhesion to the luminal surface and infiltration into the vessel wall. We therefore stained vessel sections with CD45 antibodies to identify inflammatory cells. CD45-positive cells were not detectable in unligated arteries (Fig. 8a,b). In vehicle-treated ligated arteries, we detected many CD45-positive cells adhered to the endothelium and in the enlarged subendothelial space, and some infiltrated into medial layer (Fig. 8c,f). We also observed CD45-positive cells adhered to the denuded luminal surface (Fig. 8d,g). In contrast, 5-MTP significantly reduced the number of CD45-positive cells attached to the endothelium or infiltrated into the vessel wall (Fig. 8e,h). Quantitative analysis revealed that 5-MTP decreased CD45-positive cell number from 17 ± 4 to 4 ± 2 per vessel section (*P* < 0.05 vs. vehicle group, n = 5 each group; Fig. 8i), indicating 5-MTP protects vascular endothelium from injury-induced activation/dysfunction and subsequent inflammatory cell adhesion and infiltration.

## Discussion

We demonstrated in this study a novel protective function of 5-MTP in vascular disease. We showed that CAD patients exhibit significantly lower levels of serum 5-MTP than control subjects. In a mouse model, 5-MTP protects against arterial injury-induced neointima formation through attenuating vascular injury-induced VSMC proliferation and migration and endothelial dysfunction. Furthermore, the protective effects of 5-MTP on vascular cells are predominantly mediated through p38 MAPK signaling pathway.

Various metabolites and their metabolic intermediates and byproducts participate in cellular processes. Metabolic aberrations and their downstream effects contribute to the pathogenesis of many disorders^36^. For example, succinate accumulation from ischemic insults contributes to reperfusion injury in mouse models of heart attack and stroke^37^. The tryptophan metabolite serotonin is a well-known neurotransmitter; however, a recent report showed that peripheral serotonin contributes to obesity and metabolic dysfunction^38^. On the other hand, organisms also exhibit intrinsic defense mechanisms to protect cells and tissues against pathophysiological stress and/or to repair damage. Tryptophan metabolites are involved in diverse physiological functions, including regulating circadian rhythms and reproduction^12^,^39^. In contrast to investigations in the immune and nervous systems of tryptophan metabolites^40^,^41^, study of tryptophan metabolism in vascular disease has been limited^42^,^43^,^44^,^45^. Nonetheless, it has been reported that coronary heart disease patients have lower serum tryptophan concentrations while the ratio of its metabolite kynurenine to tryptophan ratio is increased^43^. Another study showed that kynurenine pathway metabolites are associated with endothelial dysfunction markers and carotid artery intima media thickness values in patients with chronic kidney disease^46^. These studies suggest that activation of tryptophan metabolic pathways might be involved in the pathogenesis of cardiovascular disease. In line with this notion and an intrinsic defense mechanism of organisms, we found that in contrast to kynurenine, another tryptophan metabolite 5-MTP has a protective function in vascular disease. Supporting this concept, serum 5-MTP levels in humans are inversely correlated with CAD (Fig. 1a). It is tempting to speculate that reduced 5-MTP levels might contribute in part to the development of occlusive vascular disease.

5-MTP reduces intimal hyperplasia after carotid ligation (Figs 2 and 3). Consistent with the notion that development of neointima is mainly characterized by migration and proliferation of VSMCs, 5-MTP not only decreases ligation-mediated neointimal and medial VSMC proliferation *in vivo* (Fig. 3f–j) but also inflammatory cytokine IL-1β-induced VSMC proliferation and migration *in vitro* (Fig. 4e,g), suggesting a direct effect of 5-MTP on VSMCs. Intriguingly, 5-MTP did not inhibit PDGF-BB-induced VSMC proliferation (Fig. 4f), indicating 5-MTP preferentially influences IL-1β-mediated cellular effects. One early phase in the development of vascular disease involves adhesion of inflammatory cells to the vascular endothelium and subsequent infiltration into the subendothelial space and vessel wall, which are mediated through cellular adhesion molecules^47^. ICAM-1 and VCAM-1 play important roles in the firm attachment and transendothelial migration of leukocytes^47^. Consistent with this concept, we found that 5-MTP reduces ligation-induced ICAM-1 and VCAM-1 expressions (Figs 6 and 7) and the consequent inflammatory cell recruitment and infiltration (Fig. 8). Thus, 5-MTP reduces ligation-induced inflammatory responses, including endothelium detachment and denudation at an early time point (Fig. 5). These findings are in accordance with the notion that 5-MTP attenuates inflammation and the consequent reduction of proinflammatory gene expressions^9^,^10^.

Signaling pathway analyses reveal that 5-MTP attenuates IL-1β-induced VSMC proliferation via suppressing p38 MAPK and ERK1/2 activation, particularly p38 MAPK (Fig. 4h). Supporting our findings, it has been shown that p38 MAPK is activated after injury and promotes neointimal formation^32^. Interestingly, in endothelial cells 5-MTP mainly decreases IL-1β-induced p38 MAPK phosphorylation (Fig. 7e). It is of interest that in endothelial cells p38 MAPK inhibitor RWJ-67657 blocked IL-1β-induced expression of ICAM-1 and VCAM-1 at 24 h^48^. Together, these results indicate that the anti-inflammatory effects 5-MTP might be mediated predominantly through p38 MAPK pathway in vascular cells.

In conclusion, we have identified in the present study a novel protective function of 5-MTP in vascular disease. Serum 5-MTP levels are inversely correlated with CAD in humans. 5-MTP prevents VSMC proliferation and migration, endothelial damage, and the consequent intimal hyperplasia and vascular remodeling. Based on these results, 5-MTP might be a valuable lead compound for developing new strategy for preventing and/or treating vascular injury and intimal hyperplasia.

## Methods

### Patient enrollment and measurement of serum 5-MTP levels

Patients with coronary artery disease (CAD) and control subjects were enrolled in the study between September 2013 and July 2015. The Ethics Committee on Human Studies at Tri-Service General Hospital, National Defense Medical Center in Taiwan approved the study protocol (TSGHIRB # 2-102-05-104 and 2-102-05-105) and written informed consent was obtained from all participants. The study was conducted in accordance with the Declaration of Helsinki. The presence of CAD was confirmed by coronary angiography and CAD was defined as more than 50% angiographic diameter stenosis in one or more coronary arteries. CAD patients who had diabetes, hypertension, or stroke were excluded from the study. Twenty-three patients with CAD (17 males and 6 females) and 20 control subjects (10 males and 10 females) without CAD and known systemic disease were included in the study. Blood was drawn from patients prior to angiography and serum collected and stored at −80 °C until analysis. Clinical Characteristics of study participants were measured and are shown in Table 1.

5-MTP was measured by a competitive ELISA in a 96-well microtiter plate coated with polyclonal rabbit anti-5-MTP antibodies (Abcam, ab6476) using a coating buffer of 0.05 mol/L carbonate-bicarbonate (pH 9.6) at 4 °C overnight. After PBST washing and treatment with blocking buffer, mixture of 5-MTP-conjugated HRP generated by using a NH_2_ peroxidase labeling kit (Abnova) and 5-MTP standards, serum samples were added to the wells and incubated at 4 °C overnight. The wells were washed and treated with a substrate tetramethylbeuzidine at room temperature for 30 min. Reaction was stopped with 0.1N H_2_SO_4_ and the product was measured at 450 nm. The calibration curve was established by using pure 5-MTP at concentrations from 0.01 to 50 μmol/L.

### Measurement of blood pressure in mice

C57BL/6 wild-type mice (National Laboratory Animal Center, Taiwan) were used for experiments. A noninvasive tail-cuff method was used to measure systolic blood pressure (SBP) using a non-preheating MK-2000ST system (Muromachi Kikai Corp.). Conscious mice (~12-week-old male mice) were placed in special mouse holders and acclimated to the device for 10 min before measurement. A minimum of 3 serial measurements was made and the average value calculated. The SBP of each mouse was measured at baseline before IP injection of vehicle (PBS) or 5-MTP (Sigma, M4001) (100 mg/kg, 3 times a week^9^) and at different time points following initial injection. 5-MTP was dissolved in 0.05 N HCl (made in PBS) first, pH adjusted to 7.4 by NaOH, and then a stock solution of 6.5 mg/mL was prepared with PBS.

### Neointima formation model of carotid artery ligation in mice

Approximately 12-week-old male C57BL/6 wild-type mice were subjected to a neointima formation model by ligating the left common carotid artery (LCCA) as described^22^. All experimental procedures were performed in accordance with NIH guidelines (Guide for the care and use of laboratory animals) and approved by the Institutional Animal Care and Use Committee of National Health Research Institutes, Taiwan (#NHRI-IACUC-101144-A). Mice were anesthetized with tribromoethanol solution at a dose of 250 mg/kg by IP injection. After assessing the level of anesthesia by checking the pedal reflex, the LCCA was exposed through a midline incision in the neck. The LCCA was then ligated near the carotid bifurcation with a suture, skin closed, and the animals were allowed to recover from anesthesia and showed no symptoms of stroke. Following surgery, mice were treated with vehicle (PBS) or 5-MTP (100 mg/kg) by IP injection 3 times a week^9^. At indicated time points, mice were sacrificed by an overdose of tribromoethanol solution (500–750 mg/kg) by IP injection, perfused with saline, followed by 10% neutral-buffered-formalin. The contralateral right common carotid artery (RCCA, as control) and ligated LCCA were then carefully dissected, excised, and immersed in 10% formalin before processing and embedding in paraffin.

### Histological analysis and immunohistochemistry

Vessel sections (4 μm) were stained with H&E for morphology. To delineate elastin layers, we stained sections with Verhoeff’s stain (Sigma) for elastin. Three sets of sections at 150-μm intervals were used for morphometry. The intimal and medial areas were measured using NIH ImageJ software. The average intimal area, medial area, and intima-to-media ratio were calculated. To determine cellular proliferation in the injured vessel wall, mice were injected twice with BrdU (Sigma) at 16–18 h and 1–2 h before harvest. Vessel sections were stained with a monoclonal BrdU antibody (Dako) to detect proliferating cells and counterstained with hematoxylin. Immunostained positive cells were quantified as the number of positive cells divided by total number of nuclei in the neointima or media, and expressed as % of total cells.

Immunostaining was performed on sections with IL-1β antibody (R&D) to detect IL-1β expression. To detect endothelium, sections were incubated with CD31 antibody (Abcam). To examine the extent of endothelial damage, we assessed endothelial detachment and denudation. Three sets of sections at 150-μm intervals were used for morphometry. For endothelial detachment, CD31-positive endothelium that was detached from internal elastic lamina (IEL) was divided by the circumference of the lumen and multiplied by 100. For endothelial denudation, CD31-negative luminal lining was divided by the circumference of the lumen and multiplied by 100. The expressions of adhesion molecules, ICAM-1 and VCAM-1, were examined by incubating sections with ICAM-1 (R&D) or VCAM-1 antibodies (Santa Cruz), respectively, and measured by colorimetric analysis using NIH ImageJ software. To measure ICAM-1 expressions, we set a threshold just below unligated vessel staining and calculated positively stained pixel number in the endothelium^49^. The number of positively stained pixels was divided by total pixel number of endothelium. The ICAM-1 expression level is then expressed relative to unligated vessels. For VCAM-1, the number of positively stained pixels in the endothelium and media was divided by the total number of pixels and then multiplied by 100. To detect inflammatory cells, sections were incubated with an antibody against the common leukocyte antigen, CD45 (BD Pharmingen). To assess inflammatory cell accumulation, the total number of CD45-positive cells at three sets of sections at 150-μm intervals was counted and averaged.

### VSMC culture, viability, proliferation, and migration assays

Primary VSMCs were isolated from mouse aortas and cultured in DMEM as described^50^ and cells of passages 5–8 were used for experiments. To determine the effect of 5-MTP on cell viability, VSMCs were plated in 24-well plates (1.6 × 10^6^ cells/well), serum starved in quiescent medium (0.4% FBS) containing different concentrations of 5-MTP for 24 h. MTT assays were then performed and cell viability presented as a percentage of control without 5-MTP. To determine the effect of IL-1β on cell proliferation, VSMCs were plated in 24-well plates (8 × 10^3^ cells/cm^2^), serum starved for 24 h, and then treated with different concentrations of IL-1β (PeproTech) along with bromodeoxyuridine (BrdU) for 24 h. Proliferation was then assessed according to manufacturer’s instructions (Millipore). To assess the effect of 5-MTP on IL-1β-induced proliferation, VSMCs were plated, serum starved in the presence of vehicle (DMSO) or 5-MTP, and then treated with 10 ng/mL IL-1β along with BrdU for 24 h, and proliferation assessed. To determine the effects of MAP kinases on VSMC proliferation, VSMCs were pretreated with different concentrations of p38 MAPK inhibitor SB203580 (InvivoGen), SB202190 (Sigma), or MEK inhibitor U0126 (Invivogen) 30 min prior to IL-1β stimulation. BrdU incorporation assays were then performed 24 h later to assess proliferation.

To assess migration, VSMCs were serum-starved for 24 h in the absence or presence of 5-MTP (100 μmol/L) and then treated with or without IL-1β (10 ng/mL) for 24 h. Cells were placed in the upper chamber of 24-well transwell plates (Millipore, 8-μm pore size) in triplicate (10,000 cells/well). The bottom chambers were filled with quiescent medium containing PDGF-BB (Peprotech, 10 ng/mL) as a chemoattractant. After 4 h, the upper layer was scraped free of cells, membrane fixed, and stained with DAPI (Sigma). Cells that had migrated to the underside of the membrane were visualized using a fluorescent microscope. Cell number of the whole membrane was counted using ×40 images and normalized to control without IL-1β and 5-MTP treatment.

### Phosphorylation of MAP kinases and NFκB and p38 MAPK activity assays

To evaluate the effect of IL-1β and 5-MTP on MAPK and NFκB phosphorylation, VSMCs were serum starved for 24 h in the presence of vehicle (PBS) or 5-MTP (100 μmol/L), and then stimulated with or without IL-1β (10 ng/mL) for 15 min. Total proteins were then extracted for Western blotting to detect phosphorylated p38 MAPK, ERK1/2, JNK, and NFκB-p65 (Ser536) using phospho-antibodies (Cell Signaling Technology). The blots were subsequently probed with a pan-actin antibody (Millipore) to verify equivalent loading. To measure p38 MAPK activity, a p38 MAPK activity assay kit (Cell Signaling Technology, #9820) was used by immunoprecipitating active p38 with phospho-p38 antibody, followed by measuring phosphorylation of its substrate ATF2. Briefly, VSMCs were serum starved for 24 h in the absence or presence of 5-MTP (100 μmol/L), and then stimulated with or without IL-1β (10 ng/mL) for 15 min. Cell lysates were prepared and incubated with immobilized phospho-p38 antibody overnight at 4 °C. Immunocomplex kinase assays were performed at 30 °C for 30 min using ATF2 fusion protein and ATP in kinase buffer. The reaction was terminated with SDS sample buffer and the p38 activity was determined by phospho-ATF2 immunoblotting. Aliquots of cell lysates from different treatment groups were subjected to immunoblotting with p38 MAPK antibody (Cell Signaling Technology) to detect total p38 and verify equivalent amount of protein for immunoprecipitation.

### Endothelial cell culture, viability, detection of adhesion molecules, and phosphorylation of MAP kinases and NFκB

Primary human umbilical vein endothelial cells (HUVECs) (Bioresource Collection and Research Center, Hsinchu, Taiwan) were grown in endothelial basal media (Lonza), supplemented with endothelial growth media (EGM)-2 SingleQuot Kit (Lonza) containing defined supplements. Cells of passages 5–8 were used for experiments. To determine the effect of 5-MTP on HUVEC viability, cells were seeded at a density of 1.6 × 10^4^ cells per well of 96-well plate. After overnight incubation, HUVECs were treated with indicated concentrations of 5-MTP in culture medium for 24 h. MTT assays were then performed and cell viability was presented as a percentage of control without 5-MTP.

To evaluate the effect of IL-1β and 5-MTP on endothelial adhesion molecule expressions, HUVECs were plated at a density of 5 × 10^4^ cells/cm^2^ and incubated overnight. Following 24 h vehicle (DMSO) or 5-MTP (100 μmol/L) pretreatment, cells were stimulated with indicated concentrations of IL-1β for 24 h, and total proteins isolated for Western blot analysis. To detect adhesion molecules, membranes were probed with ICAM-1 (R&D) and VCAM-1 (Santa Cruz) antibodies. The blots were subsequently probed with a pan-actin antibody (Millipore) to verify equivalent loading. To examine the effect of IL-1β and 5-MTP on phosphorylation of MAP kinases and NFκB, HUVECs were pretreated with vehicle (PBS) or 5-MTP (100 μmol/L) for 24 h, and then stimulated with different concentrations of IL-1β for 15 min. Total proteins were then prepared for Western blotting. Phosphorylations of p38 MAPK, ERK1/2, JNK, and NFκB were detected using antibodies as described for VSMCs. The blots were subsequently probed with a α-tubulin antibody (GeneTex) to verify equivalent loading.

### Statistical analysis

Data are presented as mean ± S.E. of at least three independent experiments. All results were analyzed statistically by Student’s t-test. P values < 0.05 are considered statistically significant.

## Additional Information

**How to cite this article**: Ho, Y.-C. *et al*. A Novel Protective Function of 5-Methoxytryptophan in Vascular Injury. *Sci. Rep*. **6**, 25374; doi: 10.1038/srep25374 (2016).

## Figures and Tables

**Table 1:** Patient characteristics.

Characteristics	Control (n = 20)	CAD (n = 23)	*P*
Male/female	10/10	17/6	
Age (years)	59 ± 7	61 ± 10	0.33
Body mass index (kg/m^2^)	24.2 ± 3.1	24.8 ± 3.5	0.27
Systolic blood pressure (mmHg)	131 ± 4	131 ± 4	0.97
Diastolic blood pressure (mmHg)	82 ± 3	74 ± 3	0.09
Creatinine (mg/dL)	0.87 ± 0.01	1.45 ± 0.38	0.17
Glucose (mg/dL)	98 ± 3	118 ± 7	0.01
Total cholesterol (mg/dL)	208 ± 2	174 ± 10	0.02
Triglyceride (mg/dL)	110 ± 10	127 ± 14	0.36
IL-6 (pg/mL)	0.10 ± 0.07	432.8 ± 151.6	0.02
Uric acid (mg/dL)	5.4 ± 0.4	6.4 ± 0.4	0.09
Hemoglobin (g/dL)	14.1 ± 0.3	13.6 ± 0.3	0.37
Alanine aminotransferase (IU/L)	26.6 ± 4.0	21.7 ± 2.9	0.32

Data are presented as mean ± SE and were analyzed statistically by Student’s t-test. *P* values < 0.05 are considered statistically significant.
